# Restoration of intestinal function in an MPTP model of Parkinson’s Disease

**DOI:** 10.1038/srep30269

**Published:** 2016-07-29

**Authors:** L. J. Ellett, L. W. Hung, R. Munckton, N. A. Sherratt, J. Culvenor, A. Grubman, J. B. Furness, A. R. White, D. I. Finkelstein, K. J. Barnham, V. A. Lawson

**Affiliations:** 1 The Department of Pathology, The University of Melbourne, Victoria 3010, Australia; 2 Florey Institute of Neuroscience and Mental Health, The University of Melbourne, Victoria 3010, Australia; 3 The Department of Anatomy and Neuroscience, The University of Melbourne, Victoria 3010, Australia; 4 The Department of Pharmacology, The University of Melbourne, Victoria 3010, Australia; 5 Bio21 Molecular Science and Biotechnology Institute, The University of Melbourne, Victoria 3010, Australia.

## Abstract

Patients with Parkinson’s disease often experience non-motor symptoms including constipation, which manifest prior to the onset of debilitating motor signs. Understanding the causes of these non-motor deficits and developing disease modifying therapeutic strategies has the potential to prevent disease progression. Specific neuronal subpopulations were reduced within the myenteric plexus of mice 21 days after intoxication by the intraperitoneal administration of MPTP (1-methyl-4-phenyl-1,2,3,6-tetrahydropyridine) and was associated with a reduction in stool frequency, indicative of intestinal dysfunction. Oral administration of the divalent copper complex, Cu^II^(atsm), which has been shown to be neuroprotective and restore motor performance to MPTP lesioned mice, improved stool frequency and was correlated with restoration of neuronal subpopulations in the myenteric plexus of MPTP lesioned mice. Restoration of intestinal function was associated with reduced enteric glial cell reactivity and reduction of markers of inflammation. Therapeutics that have been shown to be neuroprotective in the central nervous system, such as Cu^II^(atsm), therefore also provide symptom relief and are disease modifying in the intestinal tract, suggesting that there is a common cause of Parkinson’s disease pathogenesis in the enteric nervous system and central nervous system.

Parkinson’s Disease (PD) is a neurodegenerative disorder characterized by chronic and progressive motor impairment including dyskinesia, rigidity, instability, and tremors^1^. Patients also experience significant non-motor symptoms including hyposmia, REM-sleep behaviour disorders, depression, and constipation^2^. These non-motor symptoms have recently been recognized as pre-motor features of PD and may be early markers of disease.

While the etiology of idiopathic PD is unclear it is characterized by the presence of Lewy bodies and Lewy neurites, which are primarily composed of α-synuclein, and the loss of dopaminergic neurons in the substantia nigra pars compacta (SNpc)^2^. These pathologies correlate with the subsequent motor disturbances experienced by patients. In addition to classic motor disturbances virtually all PD patients develop some level of autonomic dysfunction, including those affecting the gastrointestinal tract^3^. It is not clear whether gastrointestinal symptoms are the consequence of a loss of extrinsic innervations arising from neuronal loss in the central nervous system (CNS) or a primary consequence of pathogenesis in the enteric nervous system (ENS). However, epidemiological and histological studies suggest that gastrointestinal symptoms (constipation) and α-synuclein inclusions are present in the ENS many years before the onset of motor symptoms and inclusions occur in the CNS^4^,^5^. Furthermore recent studies highlighting the ability of α-synuclein to undergo ‘prion-like’ misfolding and aggregation^6^,^7^ are consistent with the hypothesis that disease may originate in the peripheral organs such as the ENS and progress to the CNS via the dorsal motor nucleus of the vagus where this pathological process would systematically affect the brain stem, mid- and fore-brain and eventually the cerebral cortex^4^,^8^.

The ENS is the division of the autonomic nervous system that provides intrinsic control of the gastrointestinal system^9^. The neurons of the ENS are organised into two major sets of ganglia; the myenteric plexus (MP) located between the longitudinal and smooth muscle layers, and the submucosal plexus (SMP) found in the submucosa. The function of the gastrointestinal tract is also influenced by extrinsic innervations that arise from the dorsal motor nucleus of the vagus to promote increased gut motility and sympathetic innervations from the spinal ganglia to inhibit gastric motility. The neuronal types in the ENS include primary afferent neurons, interneurons and motor neurons (inhibitory or excitatory). Most neurons involved with gastrointestinal motility are found in the myenteric plexus.

Animal models have been instrumental in our understanding of the pathogenesis of PD. Chemical induction of lesions using MPTP, rotenone or 6-OHDA; the expression of α-synuclein encoding mutations associated with familial PD; or the seeding of brain with α-synuclein have all been shown to induce motor changes and pathology consistent with PD^10^. Relatively few studies have examined the ENS, those that have show changes in neuronal populations, accumulation of α-synuclein and changes in gastrointestinal function^11^. α-synuclein has been reported to aggregate in the myenteric neurons of hA53T transgenic mice and propagate from the gut to the brain in rats following injection of human brain extracts containing aggregated α-synuclein suggesting the potential for transmission of synucleinopathy in the ENS^8^,^12^.

Current therapeutic strategies achieve symptomatic relief of the motor symptoms of PD by providing dopamine precursors, dopamine agonists, or inhibiting dopamine breakdown but do not address the underlying pathogenesis of the disorder^2^. Dopamine precursors have also been reported to improve non-motor symptoms^13^, although treatments for non-motor functions remain largely inadequate and are directed to symptom relief. Disease modifying therapies that target the underlying pathogenesis of disease offer the possibility of slowing or stopping the underlying neurodegeneration process.

Mitochondrial dysfunction, oxidative stress, inflammation and protein mishandling are likely and interrelated mechanisms of CNS pathology in patients with PD. A reactive gliosis involving astrocytes and microglial cells is observed in regions of neurodegeneration in patients with PD^14^. This may be a protective mechanism, elicited in response to cell death to clear extracellular debris and produce neurotrophic factors to support neuron survival. But it may equally contribute to pathogenesis through the production of reactive oxygen (ROS) and nitrogen species.

Cu^II^(atsm) is a copper loaded thiosemicarbazone that has been used for the diagnosis and treatment of cancer and has been shown to delay disease in several models of neurodegeneration^15^,^16^,^17^. The therapeutic efficacy of Cu^II^(atsm) has been attributed to its metal chelator activity and antioxidant/ROS scavenging capacity, both of which are likely to impact on the mechanisms of PD and has, as a consequence, the potential to offer both disease modifying and symptom relieving therapy. In support of this treatment of mouse models of PD with Cu^II^(atsm) improved motor and cognitive function and rescued nigral cell loss^15^. In the current study the effect of Cu^II^(atsm) on intestinal function and pathology in MPTP lesioned mice is described.

## Results

**Figure 1:**
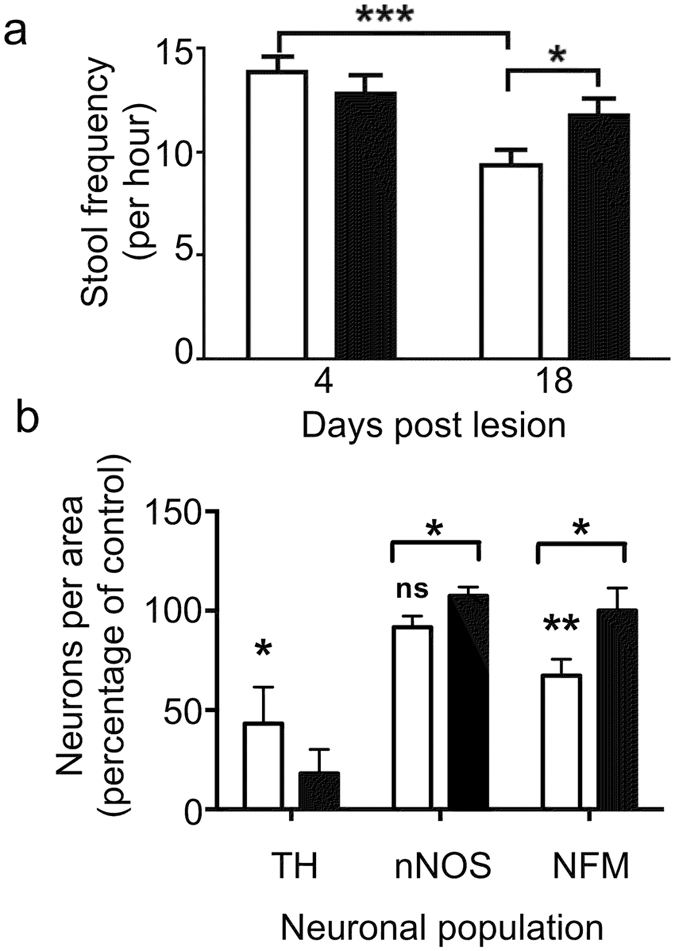
Cu^II^(atsm) restores gastrointestinal function and neuronal loss following MPTP lesioning. Following MPTP lesioning mice were treated with SSV (open bars) or Cu^II^(atsm) (closed bars) for 21 days. Stool frequency (**a**) that was significantly reduced at 18 days compared to 4 days following MPTP lesioning was restored by Cu^II^(astm) treatment in mice 18 days after post lesioning. 2way ANOVA with Bonferroni’s multiple comparisons test; ***p < 0.001 *p < 0.05; n = 20; mean ± sem. Neuronal populations (**b**) were quantified in the myenteric plexus of MPTP lesioned mice treated with SSV (open bars) or Cu^II^(astm) (closed bars). The number of neurons per field of view is expressed as a percentage of the average number of neurons in saline lesioned/SSV treated mice (control). The number of tyrosine hydroxylase (TH) and neurofilament M (NFM) immunoreactive neurons was significantly reduced in MPTP lesioned mice. The number of neuronal nitric oxide synthase (nNOS) immunoreactive neurons was not affected by MPTP lesioning. The number of NFM-IR and NOS-IR neurons was significantly increased by Cu^II^(atsm) treatment, whereas treatment had no significant affect on the number TH-IR neurons. *p < 0.05, **p < 0.01 two-tailed t-test, n ≥ 5; mean ± sem.

Intraperitoneal injection of an acute dose of the neurotoxin MPTP has been reported to cause a transient increase in colon motility 2–3 days after treatment^18^ and delayed transit and constipation 7 days after treatment^19^. To determine whether changes in gut motility occur under the current MPTP regimen one-hour stool frequency and stool weight was determined in MPTP lesioned mice (Fig. 1a). Stool frequency was significantly decreased in mice 18 days after lesion relative to 4 days post lesion and was significantly improved after 18 days of Cu^II^(atsm) treatment. In contrast stool weight was not significantly affected (data not shown). This suggests that MPTP lesions affect intestinal motility but not absorption and significantly that Cu^II^(atsm) treatment can reverse this outcome.

Enteric dopaminergic neurons are predominantly found in the SMP and MP of the upper gastrointestinal tract where the release of dopamine inhibits gut motility^20^,^21^. In mice, MPTP treatment has been shown to decrease tyrosine hydroxylase immunoreactivity (TH-IR) in the upper intestine (ileum and duodenum), but not affect TH-IR in the lower intestine (colon)^18^,^19^. To determine whether treatment with Cu^II^(atsm) can attenuate MPTP induced loss of dopaminergic neurons in the ENS of the upper intestine, whole mounts were prepared from the MP of the distal ileum and the number of TH-IR neurons were quantified. Consistent with previous reports^18^ there was a significant decrease in the number of TH-IR neurons in the MP of MPTP lesioned mice (Fig. 1b, Supplementary Figure 1) and no significant change in TH-IR in the SMP (Supplementary Figure 1). Unlike the effect of Cu^II^(atsm) on nigral neurons of the SNpc, treatment did not significantly affect the numbers of TH-IR neurons in the ENS. Thus it would appear that although TH-IR neurons of the upper intestine are affected by MPTP lesioning, the observed improvement in gut motility cannot be attributed to the restoration of TH-IR in this population after treatment with Cu^II^(atsm).

Within the ileum of the mouse approximately 30% of neurons are neurofilament medium (NFM) positive type II neurons, which also express choline acetyltransferase (ChAT) and are classified as intrinsic primary afferent neurons; and 30% are neuronal nitric oxide synthase (nNOS) positive type I neurons that represent inhibitory motor neurons and interneurons^22^. The other major types of neurons are excitatory motor neurons that express ChAT and calretinin (CalR). Excitatory cholinergic neurons are not affected in animal models of PD^18^,^21^,^23^,^24^ and reduced acetylcholinesterase (AChE) density in the gastro-intestinal tract of patients with PD was recently attributed to degeneration of vagal efferents^25^. Therefore we assessed the effect of MPTP on sensory and inhibitory neurons using NFM and nNOS staining of the ileal MP (Fig. 1b, Supplementary Figure 1).

There was significant decrease (to 67.3+/−8.3%; two-tailed t-test; p < 0.01; n ≥ 5) in the number of NFM-IR neurons in the MP of mice treated with MPTP relative to the untreated control and no significant effect on the number of nNOS-IR neurons (to 91.7+/−5.6%; n ≥ 5). Following Cu^II^(atsm) treatment there was a significant increase in the percentage of both nNOS-IR (to 107.4+/−4.5; p < 0.05; n ≥ 5) and NFM-IR (to 100.1+/−11.36; two-tailed t-test; p < 0.05; n ≥ 5) neurons relative to the MPTP lesioned mice. Cu^II^(atsm) treatment alone did not effect the immunoreactivity of any of neuronal subpopulations (Supplementary Figure 1)

**Figure 2:**
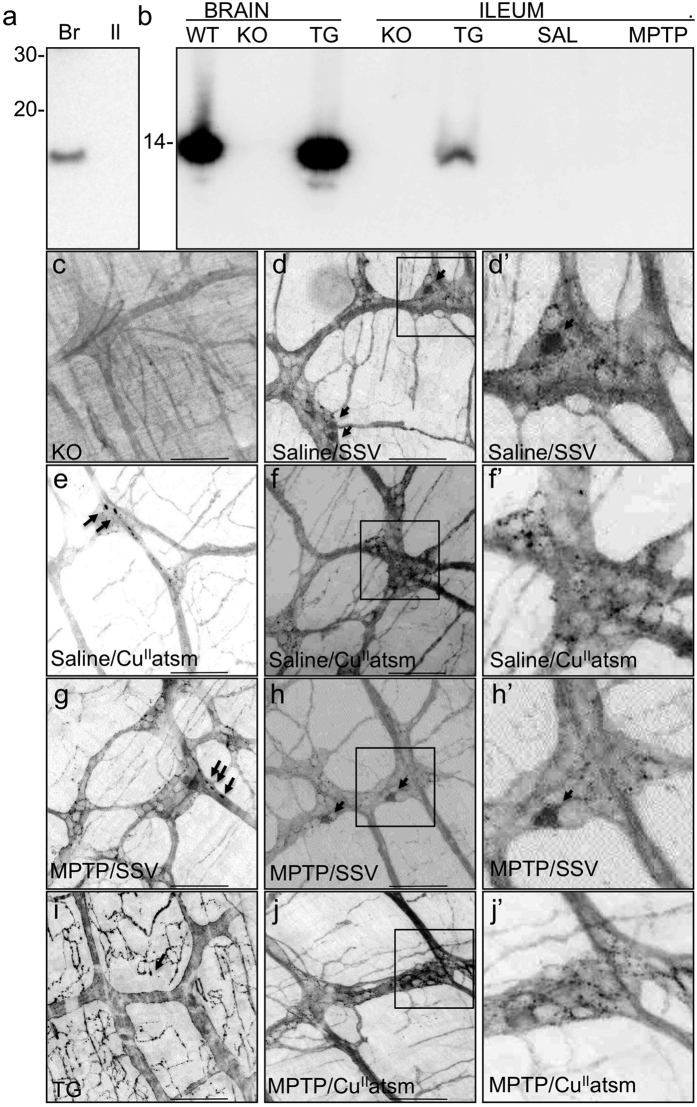
The characteristics and localisation of α-synuclein does not correlate with the therapeutic effects of Cu^II^(atsm) treatment of MPTP lesioned mice. The SDS-insoluble α -synuclein (**a**) present in the brain (Br) or ileum (Il) of hA53T transgenic mice and PBS-insoluble α -synuclein (**b**) present in the brain or ileums of hA53T transgenic (TG), α-synuclein knock-out (KO) or wild-type (WT) mice following lesioning with saline (SAL) or MPTP was detected by western immunoblot analysis of homogenates normalised for equivalent protein. α -synuclein immunoreactivity was not detected in wholemounts (**c–j**) prepared from the myenteric plexus of KO (**c**) but was present in hA53T TG (**i**) mice and saline lesioned (**d–f**) and MPTP lesioned (**g,h,j**) C57Bl6 mice, treated with SSV (**d,g,h**) or Cu^II^(atsm) (**e,f,j**). α-synuclein was observed in cell bodies (arrow heads in **d,d’,h,h’**) and as aggregates (arrows in **e,g**). Insets (**d,f,h,j**) are shown magnified x2.5 (d’,f’,h’,j’). Scale bar 100 μm.

Lewy bodies and neurites containing α-synuclein are a key pathology of PD in the CNS and have also been observed in the ENS of some patients^4^. To see if there is similar pathology in the upper intestine of mice treated with MPTP, we assessed the solubility of α-synuclein (Fig. 2). α-synuclein was detected in SDS insoluble/urea soluble fraction of brains from hA53T α-synuclein overexpressing mice but not ileums (Fig. 2a). In contrast PBS insoluble α-synuclein (Fig. 2b) could be detected in both the brains and ileum of mice overexpressing hA53T and the midbrain of WT mice but could not be detected in the ileum of wildtype saline or MPTP treated mice. Although insoluble α-synuclein was not detected in the ileum of MPTP lesioned mice, this may have reflected a limitation in the sensitivity of the method. Therefore, to further assess changes in α-synuclein induced by MPTP, immunofluorescence analysis of α-synuclein in whole mount preparations of the MP was performed.

Immunofluoresence analysis of α-synuclein in whole mounts of the MP showed the absence of α-synuclein-IR in α-synuclein knock-out (KO) mice (Fig. 2c) and abundant expression in hA53T mice (Fig. 2i) where it was predominantly located in the neurites of the tertiary plexus, with some punctate staining also observed. α-synuclein-IR was also present in the neurites of the tertiary plexus of WT mice (Fig. 2d), with expression also observed in neurites and cell bodies present within the ganglia (Fig. 2d’). There was no overt effect of MPTP treatment on α-synuclein-IR (Fig. 2g,h). Occasionally dense α-synuclein-IR was observed that may have been Lewy bodies or neurites in MPTP treated mice (Fig. 2g), however these densities were also occasionally observed in the Cu^II^(atsm) treated mice (Fig. 2e). There was a striking absence of α-synuclein-IR in cell bodies of Cu^II^(atsm) treated mice (Fig. 2e,f,j), although this was not statistically significant by quantitation (data not shown) and did not correspond with a treatment effect.

**Figure 3:**
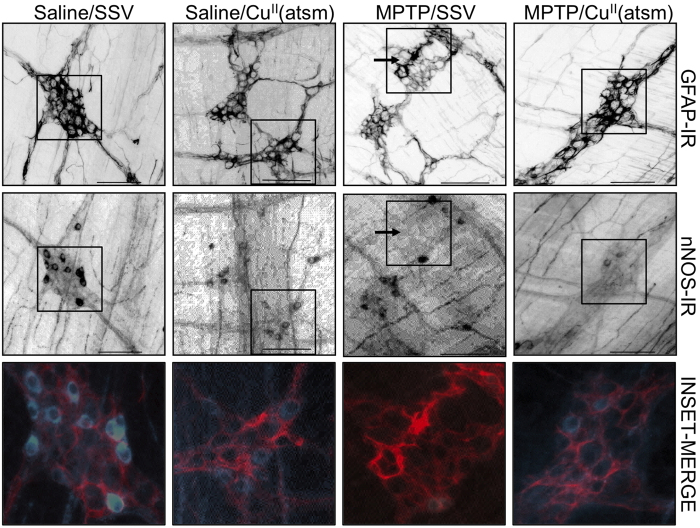
Glial cell immunoreactivity correlates with loss of nNOS-IR in MPTP lesioned mice. GFAP-IR and nNOS-IR cells were detected in whole mounts prepared from the myenteric plexus of saline or MPTP lesioned C57Bl6 mice treated with SSV or Cu^II^(atsm). In areas of glial cell distortion (arrows) there was a corresponding absence of nNOS-IR. Insets are shown as merged images of GFAP-IR (red) and nNOS-IR (blue) magnified x2.5. Note strongly immunoreactive thickened glial cell process in the MPTP/SSV image. Scale bar 100 μm.

Glial cell reactions have been observed in the SNpc in a rodent model of PD^26^ and have been reported in conjunction with the loss of nNOS-IR neurons in the ileums of mice affected with prion disease^27^. To investigate whether the enteric glial cells of the upper intestine contribute to the pathology within the ENS in this MPTP model of PD, glial fibrillary acidic protein (GFAP) staining was performed (Fig. 3). In saline lesioned mice with and without Cu^II^(atsm) treatment the myenteric ganglia show a typical morphology with enteric glial cell bodies and processes supporting neuronal subpopulations, including nNOS-IR neurons. In MPTP-lesioned mice there were areas where the GFAP-IR revealed shortened intensely labelled glial cell processes which corresponded with regions lacking nNOS-IR neurons (Fig. 3). In Cu^II^(atsm) treated MPTP-lesioned mice, the intensity of GFAP-IR was reduced relative to MPTP-lesioned preparations and longer glial cell processes surrounded nNOS-IR neurons. Thus although we did not detect a significant decrease in the number of nNOS-IR neurons (Fig. 1b) in MPTP-lesioned mice there were areas of ganglia in which the numbers of nNOS-IR neurons were reduced and glial cells were more strongly immunoreactive and had shortenened, thickened processes (Fig. 3). These effects were attenuated by treatment with Cu^II^(atsm).

**Figure 4:**
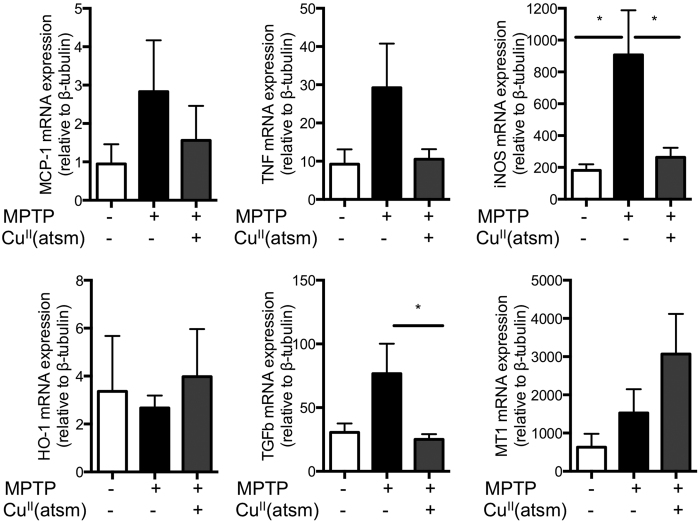
The expression of pro-inflammatory molecules correlates with therapeutic effects of Cu^II^(atsm) treatment of MPTP lesioned mice. The expression of pro-inflammatory (MCP-1, TNF, iNOS) and anti-inflammatory (HO-1, TGFb) genes and the metallothionen gene (MT-1) were assessed in mRNA isolated from segments of ileum prepared from saline and MPTP lesioned mice treated with SSV or Cu^II^(atsm). The cycle threshold is shown relative to β-tubulin. *p < 0.05 two-tailed t-test, n = 5–6, mean ± SEM.

Changes in GFAP-IR suggest that inflammation may be contributing to the pathology observed in the ENS of MPTP lesioned mice. RT-PCR analysis for markers of inflammation revealed a significant increase in the expression of mRNA for the pro-inflammatory molecule iNOS (inducible nitric oxide synthase) in MPTP lesioned mice that was attenuated by Cu^II^(atsm) treatment (Fig. 4). There was no significant effect of MPTP lesions or Cu^II^(atsm) treatment on the collective expression of the other pro-inflammatory molecules investigated (monocyte chemoattractant protein-1; MCP-1, tumour necrosis factor alpha; TNFα), although the increased expression of these molecules following MPTP treatment did correlate significantly with the expression of iNOS (Supplementary Figure 2). The expression of mRNA for the anti-inflammatory molecule HO-1 (heme oxygenase 1) was unaffected by MPTP lesioning and Cu^II^(atsm) treatment whereas TGFb (transforming growth factor beta) mRNA expression was significantly decreased, relative to MPTP-lesioned mice, following Cu^II^(atsm) treatment. MT-1 (metallothionen 1) is a protein that maintains metal ion homeostasis and therefore protects cells from metal ion induced oxidative stress. It has been proposed to mitigate the oxidative damage caused by MPTP treatment, however in this study the expression of MT-1 was not significantly changed in either MPTP or MPTP/Cu^II^(atsm) treated mice.

## Discussion

Non-motor symptoms affecting patients with Parkinson’s disease are prodromal to the motor dysfunction that typifies the disease. Cu^II^(atsm) was recently reported to improve motor and cognitive function and rescued nigral cell loss in animal models of Parkinson’s disease^15^. In the current study administration of Cu^II^(atsm) to MPTP lesioned mice restored intestinal function, rescued the loss of neurofilament positive neurons in the myenteric plexus of the ileum and reduced markers of inflammation. These results extend the disease modifying effects of Cu^II^(atsm) from motor function in the CNS to non-motor function in the ENS and hold the promise for effective therapeutics that could limit disease progression.

Central to the motor-symptoms in Parkinson’s disease is the progressive loss of dopaminergic neurons in the SNpc. Dopaminergic neurons, identified by the production of tyrosine hydroxylase (TH-IR), are reduced in the SNpc of mice following MPTP lesioning. We report a similar reduction in the proportion of TH-IR neurons in the MP of the ENS of the ileum of mice following MPTP lesioning. A similar loss of TH-IR dopaminergic neurons has not been detected in the MP (stomach, duodenum or colon;^28^) or SMP (colon;^29^) of the ENS of patients with PD; however, these studies did not assess TH-IR in the ileum. Variation in the distribution of TH-IR neurons along the gastrointestinal tract and the apparent differences in the susceptibility of TH-IR neurons in different regions of the gastrointestinal tract or indeed different plexuses (as reported here) in toxin-induced models PD may explain region specific loss of this neuronal population. It therefore remains to be determined whether the reduced TH-IR neurons reported here in the MPTP model is tissue (ileum) or model (MPTP) specific.

Cu^II^(atsm) has been shown to improve motor performance and rescue the number of predominantly dopaminergic nigral neurons in the SNpc of MPTP lesioned mice^15^. In contrast, Cu^II^(atsm) treatment did not restore the TH-IR population in the ENS. While it is not clear why TH-IR neurons of the ENS were not similarly rescued by Cu^II^(atsm) it does seem that the loss of these cells is unlikely to be responsible for the reduced stool frequency observed in MPTP lesioned mice even though dopamine may play an important role in gastrointestinal function by inhibiting gastrointestinal motility^30^.

In contrast to dopaminergic neurons, NFM-IR neuron numbers were significantly reduced in the MP of mice following MPTP lesioning. NFM-IR identifies intrinsic primary afferent neurons (IPANs) in the mouse intestine^22^. IPANs, also referred to as AH or Dogiel type II neurons, are the first neurons in reflex pathways that control the movements of the intestine; they respond to physiological stimuli such as distension and chemical changes in the lumen^31^. Loss of these neurons would be predicted to diminish propulsive reflexes that are initiated by fecal distension in the colon. Thus changes in these neurons in response to MPTP could be responsible for the changes in stool frequency reported here and importantly the restoration of this population following Cu^II^(atsm) treatment can explain the observed improvement in gut motility. Neurofilament immunoreactivity also marks IPANs in human intestine^32^. A re-examination of human intestine from PD patients could determine whether similar changes occur in both species. It will be important to investigate the molecular pathways through which IPANs are affected by MPTP. This may arise indirectly from inflammation, to which IPANs are particularly sensitive^33^,^34^.

nNOS-IR neurons are reduced in the distal ileum of rats bearing nigrostriatal lesions caused by 6-hydroxy-dopamine^35^,^36^ and has been reported for nNOS-IR neurons of the SNpc in MPTP lesioned mice^37^. Although nNOS-IR was not significantly reduced in the MP of MPTP lesioned mice, reported here, the number of nNOS-IR neurons was specifically reduced in regions where the intensity of GFAP-IR was increased and glial cell processes were shortened. A similar observation was observed in the MP of mice with terminal prion disease, suggesting that neuronal loss coupled with glial cell reactivity may be a common phenomenon associated with ENS degeneration^27^. Nitrosative stress is one of many factors that may contribute to the pathogenesis of PD and is also a significant player in enteric neuropathies^38^. Cu^II^(atsm) is reported to be a highly effective scavenger of peroxynitrite a reaction oxygen species caused by nitrosative stress in cells where nitric oxide is produced by NOS^15^. Therefore, the treatment effect of Cu^II^(atsm) may be through the reduction of reactive oxygen species produced through the action of nNOS and iNOS. Inhibition of nNOS and/or iNOS has been reported to attenuate the neurotoxic effects in the striata of MPTP lesioned mice^39^.

Neuroinflammatory changes including gliosis and changes in inflammatory cytokine expression are observed in the brain and CSF of patients with Parkinson’s disease and may contribute to disease progression (reviewed^40^). Increased expression of pro-inflammatory molecules and increased GFAP expression has also been reported in the colon of PD patients^41^. We observed a significant increase in the expression of iNOS in MPTP lesioned mice, which was attenuated in mice receiving Cu^II^(atsm) treatment. Increased expression of iNOS reflects a pro-inflammatory state with the production of NO acting to further exacerbate inflammation and produce reactive nitrogen species such as peroxynitrite. As further evidence of a pro-inflammatory environment there was a significant correlation between iNOS expression and TNF and MCP-1 expression in MPTP lesioned mice (Supplementary Figure 2). Although the increased expression of neither of these molecules was in itself significantly increased this may reflect variation in the intensity of the inflammatory response in individual animals. Increased expression of iNOS was recently reported to be toxic to myenteric neurons in a TBS-induced model of IBD^42^, thus the increased expression of iNOS may be a mechanism of inflammation induced neuronal loss.

Cu^II^(atsm) treatment significantly decreased the expression of TGFb detected in MPTP lesioned mice. This observation is also consistent with protection of the ENS by attenuation of proinflammatory monocytes in MPTP lesioned mice^43^,^44^. TGFb is produced by enteric glial cells in response to inflammatory stimuli and has been reported to promote neuronal plasticity and differentiation in the ENS^45^. TGFb has also been shown to be elevated in PD affected brain regions^46^ and *in vitro* has been shown to promote the survival of dopaminergic neurons and prevent MPP + induced toxicity^47^,^48^,^49^. The upregulation of this molecule in response to MPTP lesioning supports the role of an inflammatory environment in promoting gastrointestinal dysfunction and may further reflect attempts to regenerate lost neurons in MPTP lesioned mice. The decrease in TGFb expression following Cu^II^(atsm) may reflect the reduced inflammatory environment and perhaps explain the failure of this compound to protect TH-IR, dopaminergic neurons from MPTP lesioning.

Aggregation of α-synuclein in Lewy bodies and neurites is a core pathology in the CNS of patients with PD. Aggregates of α-synuclein can be detected in the ENS of patients with PD^4^,^50^ and have been shown experimentally to propagate from the gastrointestinal tract to brain^8^. In the current study there was no discernable affect on α-synuclein expression in ENS that could be directly attributed to the affects of MPTP. None-the-less there was a striking absence of localisation of α-synuclein in cell bodies following Cu^II^(atsm) treatment which may have attenuated undetectable effects of MPTP on α -synuclein localisation.

In light of these observations we propose the following model for ENS degeneration and dysfunction following MPTP treatment:

MPTP treatment leads to an increase in oxidative stress, which leads to the production of peroxynitrite in nNOS producing neurons and reactive oxygen species in TH producing cells. In addition to dysfunction caused by the direct loss of these neurons, injury of these cells results in reactivity of enteric glial cells and the induction of inflammation, including the expression of iNOS. IPANs (NFM-IR neurons) react to mediators of inflammation leading to reduced propulsive reflexes. Cu^II^(atsm) treatment restores gastric motility by scavenging peroxynitrite. This protects nNOS-IR neurons from injury and therefore does not trigger enteric glial cell reactivity and subsequent inflammation and dysfunction of NFM-IR IPANs.

## Methods

### Animal experiments

MPTP lesioning of C57BL/6 mice was performed as previously described^15^. Briefly 4-month-old C57BL/6 male mice were injected intraperitoneally with four doses of MPTP (Sigma-Aldrich), 10 mg/kg at 2-h intervals, the total dose per mouse being 40 mg/kg which has been shown to cause a 50% reduction in nigral neurons^15^. One day after lesioning mice were treated by oral gavage with 30 mg/kg diacetylbis(*N*(4)-methylthiosemicarbazonato) copper(II) (Cu^II^(atsm)) or standard suspension vehicle (SSV; NaCl, 0.9% wt/vol; carboxymethyl cellulose, 0.05% wt/vol; benzyl alcohol, 0.05% vol/vol; Tween 80, 0.04% vol/vol) via oral gavage daily for 21 d. In addition to the toxin-injected mice, a cohort of mice were sham lesioned with saline, and then orally gavaged with Cu^II^(atsm) or SSV for the duration of the trial.

Gastrointestinal function was assessed by stool frequency 4 and 18 days after MPTP or sham lesioning. Animals were deprived of food overnight. Following oral gavage, mice (n = 20) were given access to food for 2 hours prior to the test. Animals were placed in clean separate boxes for 1 hour and then returned to their home cages. Stools were collected, counted and weighed before being dried overnight at 65 °C to enable assessment of dry stool weight.

Animals were euthanised by an overdose of sodium pentobarbitone (100 mg/kg Lethobarb; Jurox), and perfused via the heart with cold 0.1 M PBS (Sigma-Aldrich), pH 7.4. Fresh segments of intestine were removed from each animal and placed in phosphate-buffered saline (PBS: 0.15 M NaCl in 0.01 M sodium phosphate buffer, pH 7.2), containing the L-type calcium channel blocker nicardipine (0.01% v/v in PBS; Sigma, Castle Hill, Sydney, Australia), to inhibit tissue contraction. The content of each segment was purged with PBS/nicardipine. Pieces of each segment were frozen in liquid nitrogen and stored at −80 °C for Western immunoblot analysis or prepared as wholemounts. Segments of ileum were also prepared from α-synuclein knock-out (KO) and transgenic (TG) mice as negative and positive controls, respectively, for wholemount and western immunoblot analysis of α-synuclein expression. The transgenic mice overexpresses human α-synuclein with the A53T mutation driven by the mouse prion promoter and were maintained as a homozygous TG line on a mixed C57Bl/6 × C3H background^51^. The knock-out line lacked endogenous mouse α-synuclein expression^52^.

### Tissue preparation and analysis

Wholemounts were prepared from segments of ileum as previously described^27^. Briefly, the segments of ileum were pinned to balsa-wood sheets for fixation in 2% formaldehyde plus 0.2% picric acid in 0.1 M sodium phosphate buffer, pH 7.2, at 4 °C overnight and cleared of fixative by 3 × 10 min washes in dimethyl sulphoxide followed by 3 × 10 min washes in PBS. Fixed tissue was stored at 4 °C in PBS containing sodium azide (0.1%). SMP preparations were obtained by removing the mucosa and separating the submucosa from the circular muscle layer. MP preparations were obtained by removing the mucosa, SMP and the circular muscle layer. Wholemounts were incubated with antibodies (Table 1), overnight at 4 °C. The wholemounts were then washed (3 × 10 min) in PBS before incubation with secondary antibodies (Table 2) for 1 h at RT. Preparations were given three subsequent 10 min washes in PBS and then mounted on glass slides, using non-fluorescent mounting medium (Dako).

Preparations were analysed by a Nikon Eclipse TE2000-E epi-fluorescence microscope. Fluorophores were visualised by using a 360-nm excitation filter and 435–485-nm emission filter for Alexa 350, and 535-nm excitation and 590-nm emission long pass filter for Alexa 594. Areas of interest were captured using a Roper Scientfic Black and White camera. MP n = 5–7, SMP n = 4–6.

The number of cells per field of view (x10 objective) were quantified and normalized to the untreated (saline/SSV) samples. A minimum of 8 fields of view was counted. For quantitation of GFAP expression images were imported into Image J.

Homogenates were prepared from frozen segments of ileum for Western immunoblot analysis of α-synuclein expression. Homogenates (10% w/v) were prepared in Dulbecco’s PBS (Gibco) supplemented with *e*complete protease inhibitors (Roche) using a dounce homogeniser and protein concentration determined by BCA assay. To detect the SDS-insoluble fraction equivalent protein was prepared in PBS supplemented with 5% SDS and centrifuged at 100,000 × g for 30 minutes. The SDS-insoluble pellet was then resuspended in 8% SDS/8M urea. To detect the PBS insoluble fraction, equivalent protein prepared in PBS was centrifuged at 10,000 × g for 30 minutes. The PBS insoluble pellet or urea solubilised pellet were prepared in sample buffer (Invitrogen) supplemented with 5% β-mercaptoethanol and electrophoresed on a 4–12% NuPAGE Bis/Tris gel in MES buffer, transferred to a PVDF membrane and probed using rabbit polyclonal antibody raised to residues 116–131 of α-synuclein (Table 1).

### Gene expression analysis using qPCR

Total RNA was isolated from segments of ileum using the RNaqueous Kit (Life Technologies). Isolated RNA (10 μg) was DNAse treated (Turbo DNA free; Ambion) and DNAse-treated RNA (2 μg) was reverse transcribed using the High Capacity cDNA kit (Life Technologies). TaqMan gene expression assays for TUBA3, CCL2/MCP-1, TGFb, TNF, MT-1, iNOS, HO-1 were purchased from Life Technologies (Mm00833707_mH, Mm00441242_m1, Mm01178820_m1, Mm00439614_m1, Mm00496660_g1, Mm00440502_m1, and Mm00516005_m1, respectively) and qRT-PCR was performed as previously described^53^. Each 10 μl reaction mix consisted of 0.5 μl of Taqman Gene Expression assays containing FAM-labelled probes, 5 μl of 2x TaqMan Gene Expression Master mix and 4 μl of cDNA template (diluted 1:4). Duplicate reactions were performed using the LightCycler 480 (Roche) using the following conditions: 2 min at 50 °C, 10 min at 95 °C, followed by 45 cycles of 15 s at 95 °C, and 60 °C for 1 min. Cycle threshold (Ct) values were calculated as the lowest cycle number producing an exponential increase in PCR product amplification. Delta Ct method was used for normalization of expression relative to β-tubulin.

### Ethics statement

All animal procedures were approved by the Florey Institute animal ethics committee (08-023,14-023) and were performed in accordance with the National Health and Medical Research Council guidelines.

### Statistical analysis

Statistical analysis was carried out using GraphPad Prism 6 statistical software. Comparison of normally distributed pairs was performed using a two-tailed t-test. For multiple comparisons one-way ANOVA was performed. Graphs show mean ± s.e.m, unless otherwise stated.

## Additional Information

**How to cite this article**: Ellett, L. J. *et al*. Restoration of intestinal function in an MPTP model of Parkinson’s Disease. *Sci. Rep*. **6**, 30269; doi: 10.1038/srep30269 (2016).

## Supplementary Material

Supplementary Information

## Figures and Tables

**Table 1:** Primary antibodies used for immunohistochemistry.

Tissue antigen	Host species	Dilution	Source/reference
TH (tyrosine hydroxylase)	Sheep	1:500	Millipore AB1542
nNOS (neuronal nitric oxide synthase)	Sheep	1:2000	Aldecoa, I. *et al*.^54^
NFM (neurofilament medium) 145 kDa	Rabbit	1:200	Millipore AB1987
GFAP (glial fibrillary acidic protein)	Rabbit	1:5000	Dako Z033429
α-synuclein	Rabbit	1:250	Ab 97/8 raised to α-synuclein residues 116–131 (Culvenor, J. G. *et al*.^55^)

**Table 2:** Secondary antibodies and labels.

Antibody	Dilution	Source
Donkey anti-rabbit Alexa Fluor^®^ 594	1:500	Life Technologies A-21207
Donkey anti-sheep Alexa Fluor^®^ 350	1:500	Life Technologies A-21097
