# Supplementary material for: Restoration of intestinal function in an MPTP model of Parkinson’s Disease
Source: Sci Rep. 2016 Jul 29;6:30269. doi: 10.1038/srep30269 (PMC4965866; doi:10.1038/srep30269)
Supplement: Supplementary Information [file srep30269-s1.pdf]

Supplementary Figures
Restoration of intestinal function in an MPTP model of Parkinson's Disease.

L. J. Ellett, L. W. Hung, R. Munckton, N. A. Sherratt, J. Culvenor, A. Grubman, J. B. Furness, A. R. White, D. I. Finkelstein, K. J. Barnham, V. A. Lawson

Supplementary Figure S1

Neuronal subpopulations in wholemount preparations of ileum from MPTP lesioned and Cu^{II}(atsm) treated mice.

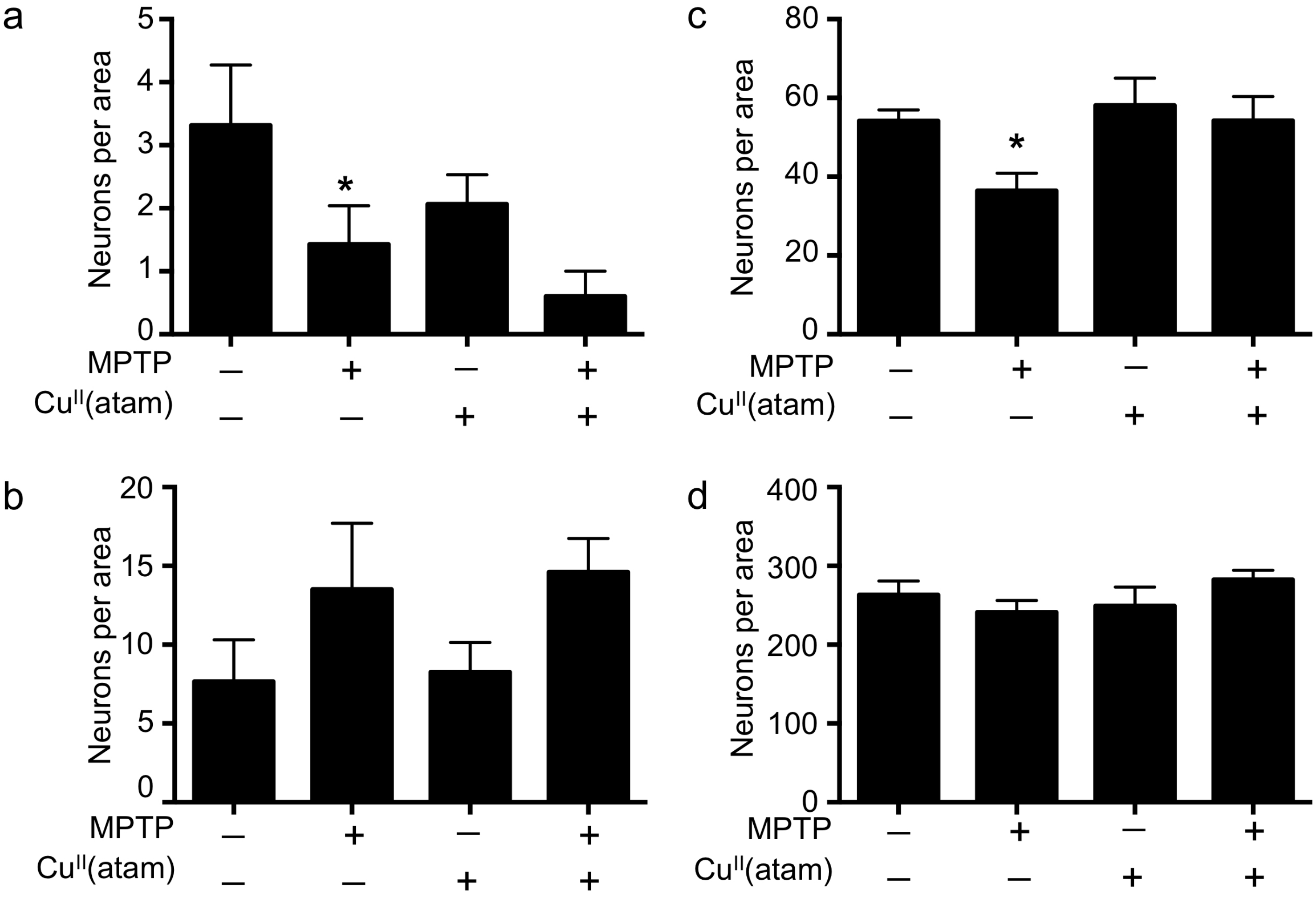

TH-IR in the MP (a) and SMP (b), NFM-IR in the MP (c) and nNOS-IR in the MP (d) of mice lesioned with MPTP and treated with Cu^{II}(atsm) are shown as number of neurons per area. *$p<0.05$ compared with untreated (Saline/SSV) using one-way ANOVA with Dunnett's multiple comparison test ($n\geq5$, mean ± sem).

Supplementary Figure S2
Correlation of pro-inflammatory marker expression in the ileum of MPTP-lesioned mice.

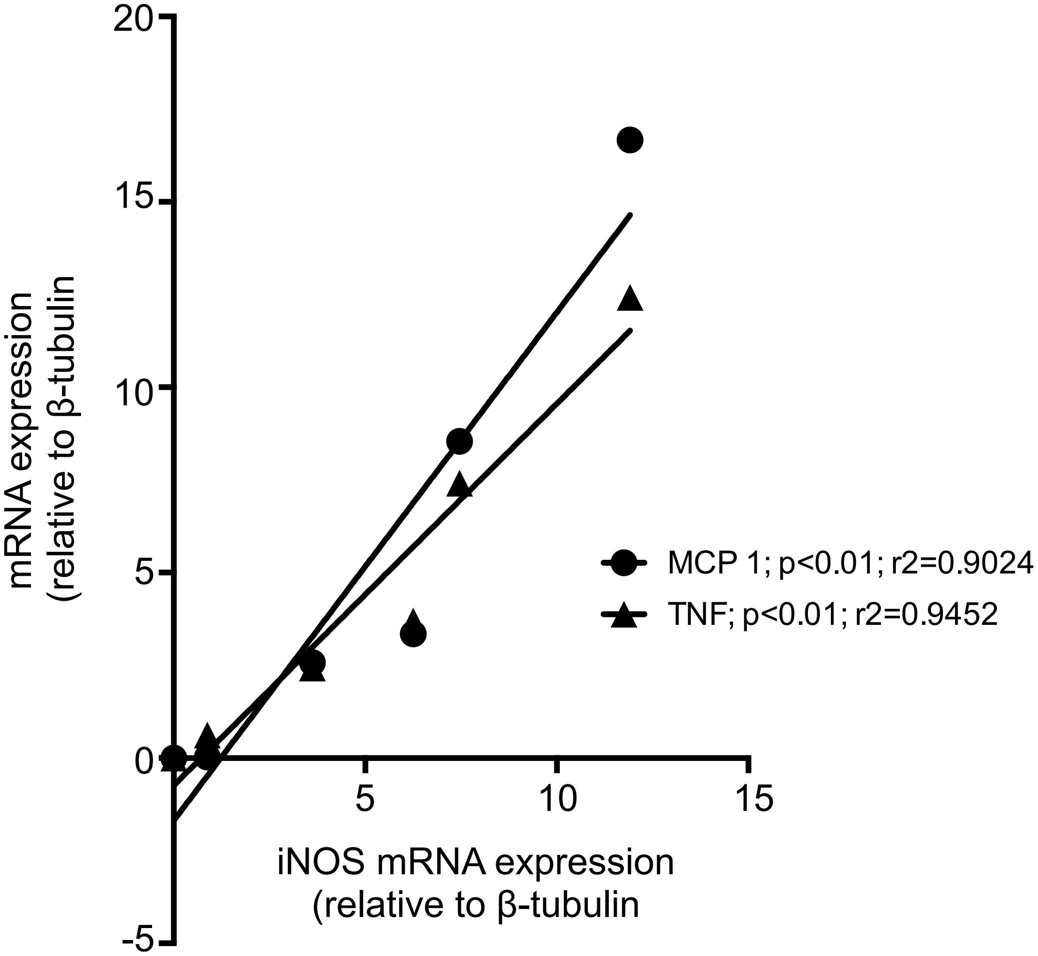

Analysis of the relative pro-inflammatory molecule expression indicated that there was a significant correlation between the expression of iNOS mRNA per mouse relative to pro-inflammatory markers MCP1 and TNF.
